# Heart rate variability changes at 2400 m altitude predicts acute mountain sickness on further ascent at 3000–4300 m altitudes

**DOI:** 10.3389/fphys.2012.00336

**Published:** 2012-08-30

**Authors:** Heikki M. Karinen, Arja Uusitalo, Henri Vähä-Ypyä, Mika Kähönen, Juha E. Peltonen, Phyllis K. Stein, Jari Viik, Heikki O. Tikkanen

**Affiliations:** ^1^Unit for Occupational Health, Department of Health Sciences, University of TampereTampere, Finland; ^2^Department of Sports and Exercise Medicine, Institute of Clinical Medicine, University of HelsinkiHelsinki, Finland; ^3^Department of Biology of Physical Activity, University of JyväskyläJyväskylä, Finland; ^4^Department of Clinical Physiology, University of Tampere and Tampere University HospitalTampere, Finland; ^5^Division of Cardiology, Washington University School of MedicineSt. Louis, MO, USA; ^6^Department of Biomedical Engineering, Tampere University of TechnologyTampere, Finland; ^7^Clinic for Sports and Exercise Medicine, Foundation for Sports and Exercise MedicineHelsinki, Finland

**Keywords:** extreme altitude, altitude illness, heart rate variation, mountaineering

## Abstract

**Objective:** If the body fails to acclimatize at high altitude, acute mountain sickness (AMS) may result. For the early detection of AMS, changes in cardiac autonomic function measured by heart rate variability (HRV) may be more sensitive than clinical symptoms alone. The purpose of this study was to ascertain if the changes in HRV during ascent are related to AMS. **Methods:** We followed Lake Louise Score (LLS), arterial oxygen saturation at rest (R-SpO_2_) and exercise (Ex-SpO_2_) and HRV parameters daily in 36 different healthy climbers ascending from 2400 m to 6300 m altitudes during five different expeditions. **Results:** After an ascent to 2400 m, root mean square successive differences, high-frequency power (HF_2 min_) of HRV were 17–51% and Ex-SpO_2_ was 3% lower in those climbers who suffered from AMS at 3000 to 4300 m than in those only developing AMS later (≥5000 m) or not at all (all *p* < 0.01). At the altitude of 2400 m RMSSD_2 min_ ≤ 30 ms and Ex-SpO_2_ ≤ 91% both had 92% sensitivity for AMS if ascent continued without extra acclimatization days. **Conclusions:** Changes in supine HRV parameters at 2400 m were related to AMS at 3000–4300 m Thus, analyses of HRV could offer potential markers for identifying the climbers at risk for AMS.

## Introduction

When ascending to high altitude, the body needs to acclimatize to low atmospheric pressure and hypoxic hypoxia (Hackett and Roach, 2001). If the adaptation process fails due to too rapid ascent rate or susceptibility of the climber, one or more of three illnesses may result: acute mountain sickness (AMS), high altitude cerebral edema (HACE) or high altitude pulmonary edema (HAPE). AMS is the most common of these problems affecting 25% of those who ascend to altitudes of 1850–2750 m (Honigman et al., 1993), 42% at altitudes of 3000 m (Hackett and Roach, 2001) and as many as 75% among those attempting Mount Kilimanjaro (5984 m) (Karinen et al., 2008). AMS is a non-specific syndrome characterized by the presence of headache and at least one of the following: gastrointestinal symptoms (loss of appetite, nausea, and vomiting), insomnia, dizziness, and weakness or fatigue. It is a self-limiting syndrome, which usually resolves in 1–2 days if properly treated (rest, descent to lower altitude, medication etc.) but may sometimes progress to more serious altitude illnesses such as HAPE and HACE (Hackett and Roach, 2001; Imray et al., 2011).

The exact mechanism causing AMS is unknown, but a marked increase in peripheral sympathetic activity is a common feature of AMS (Kamimori et al., 2009) and may be involved in the pathogenesis of HAPE (Mazzeo et al., 1998; Duplain et al., 1999; West, 2004). Heart rate variability (HRV) reflects sympathetic and parasympathetic cardiac autonomic nervous system regulation. Several studies have shown a transient reduction in parasympathetic and increased sympathetic activity during acute exposure to hypobaric hypoxia (Zuzewicz et al., 1999; Sevre et al., 2001; Saito et al., 2005; Hainsworth et al., 2007) which tended to be reversed with acclimatization (Cornolo et al., 2004).

There is a need for a non-invasive, specific and convenient method under field conditions for the detection of inadequate acclimatization and impending AMS. The arterial oxygen saturation (SpO_2_) measurement is useful in anticipating AMS (Roach et al., 1998; Karinen et al., 2010) but use of pulse oximetry under field conditions is susceptible to many disruptive factors e.g., temperature (Luks and Swenson, 2011). Reduction in parasympathetic and increased sympathetic activity during acclimatization and AMS at 3180–4559 m altitudes have been shown in several studies (Loeppky et al., 2003; Lanfranchi et al., 2005; Chen et al., 2008; Huang et al., 2010). To the best of our knowledge, studies on HRV conducted in the field at extreme altitudes (>5000 m) are still lacking, despite two studies at simulated altitude (Yamamoto et al., 1993; Wille et al., 2012). Therefore the purpose of this study was to evaluate whether HRV is related to AMS during ascent and provides new information on the changes in cardiac autonomic function as measured by HRV and not limited to altitudes between 2400 and 5000 m, which are most frequent among climbers, but also at extreme altitudes above 5000 m under field conditions.

## Materials and methods

The study group consisted of participants in four different expeditions to Denali (Denali 1, Denali 2), Shisha Pangma and Mount Everest. All subjects were informed about the objective of the study and the experimental protocol. The Ethics Committee of Tampere University Hospital, Finland, approved the study protocol, and all subjects gave informed consent prior to the measurements, as stipulated in the Declaration of Helsinki. Thirty six different healthy volunteers (2 women and 34 men) with an age range of 24–45 years participated in this study. Subjects' mean age was 32 (SD 6) years, body mass index (BMI) 25 (3) kg/m^2^ and maximal oxygen uptake ($\dot{V}{\text{O}}_{2\max}$) 59 (7) ml/kg/min. None of the subjects had been exposed to an altitude above 1000 m within the 6 months prior to this study. They were all lowland dwellers and recreational mountaineers, and during the expeditions none had taken acetazolamide as an AMS prophylaxis. Two climbers were taking regular medication for asthma but no one was using oral steroids, salmeterol, sildenafil or nifedipin. All expeditions were organized in April-May. Ascent profiles were similar at sleeping altitudes, but three expeditions had faster and two slower ascent rate. Before the expeditions, all members underwent a medical examination including resting ECG and flow volume spirometry. To examine their exercise responses and to measure maximal oxygen uptake ($\dot{V}{\text{O}}_{2\max}$), they performed an incremental clinical exercise test on a cycle ergometer (Ergoline 800S, Ergoline GmbH, Bitz, Germany). They started cycling at 20 W and work rate was increased stepwise 20 W/min up to volitional fatigue. A 12-lead ECG was obtained at rest before the exercise, and during the exercise test. All subjects had a normal EKG and none developed arrhythmias during the exercise test.

## Data collection during ascent

Autonomic cardiac function was assessed by analysis of R-R intervals (RRI). In power spectral analysis of HRV total variability of RRI is divided into low and high frequency bands. High frequency power (HF power, 0.15–0.40 Hz) reflects cardiac parasympathetic modulation and is influenced by respiration. Low frequency power (LF power, 0.04–0.15 Hz) reflects both cardiac sympathetic and parasympathetic modulation. The ratio of LF and HF powers (LF/HF) has been reported to reflect sympathovagal balance. RMSSD is the square root of the mean squared differences of successive RRI and it is considered to be an index of cardiac parasympathetic modulation (Task Force, 1996).

In the present study, HRV and SpO_2_ data were collected every day during ascent at altitudes of 2400, 3000, 3500, 4300, 5000, and 5300 m in all expeditions, and up to 5600 and 6300 m at Shisha Pangma. Data collection was continued until the base camp of each mountain was reached. Measurements were done every morning after 7–8 h night rest at that altitude by heart rate monitors (Suunto T6, Suunto, Vantaa, Finland or S810, Polar Electro, Kempele, Finland). HR recordings were made in the supine position, after at least 15 min of rest in the same position while each subject laid quietly and breathed spontaneously. Then subjects started their HR monitors and continued lying down for 3 min. The last 2 min were chosen for data analysis. According to the Task Force of the European Society of Cardiology and the North American Society of Pacing and Electrophysiology (Task Force, 1996), the preferred duration of ECG recordings for computing short-term HRV components is 5 min. The recording should last at least 10 times the wavelength of the lowest frequency bound of the investigated component (Task Force, 1996). Thus a recording of approximately 1 min is needed to assess the HF and approximately 2 min are needed to address the LF component. In our study the duration of stationary RRI time series was 2 min, which should be adequate for the determination of the HRV components evaluated (Koskinen et al., 2009). The arterial oxygen saturation at rest (R-SpO_2_) was measured by finger oximetry (Nonin Medical, Onyx 9500, Plymouth MN, USA) after 15 min rest while the subject was seated for an additional 2 min. During this period, R-SpO_2_ was observed four times at 15 s intervals and the average R-SpO2 of these was recorded for data analysis. The arterial oxygen saturation during exercise (Ex-SpO_2_) at altitude was measured with a standard walking test (Karinen et al., 2010). The climber walked and controlled his speed with the HR monitor so that HR was approximately 150 beats/min during 3–5 min walking. Temperature varied between +20 and −15°C and hands were covered by mittens during walking, which ended inside a tent. The inside temperature was mostly between +5 and 20°C because of extensive solar radiation or a stove. Immediately after the cessation of walking, SpO_2_ was measured in sitting position by finger oximetry four times at 5 s intervals during the first 15 s of recovery starting immediately after stopping the exercise test. The average of these values was recorded as Ex-SpO_2_ for data analysis. We observed that SpO_2_ decreased 15 s after the exercise began. During the first 15 s the SpO_2_ values remained very steady, varying just 0–1% unit, thus quite accurately reflecting also SpO_2_ during exercise.

## Data analysis

Data processing and analysis were performed after the expeditions by Polar Precision Performance software (version 3.02.007) and Kubios software (version 2.0) (Niskanen et al., 2004). Areas of ectopy or artifact were identified and fixed by manual or automatic error correction. Corrected segments were less than 5% of the analyzed data. Analysis of HRV was done for stationary 2 min data segments. The power spectra were quantified by measuring the area in two frequency bands. The HF_2 min_ power was calculated for frequency band 0.15–0.40 Hz and LF_2 min_ power for frequency band 0.04–0.15 Hz.

After the HRV and SpO_2_ measurements subjects were scored according to the Lake Louise scoring (LLS) AMS system, which include a self-report questionnaire related to the presence and severity of symptoms and a clinical assessment (Hackett and Oelz, 1992). The LLS was obtained by adding the score of the clinical section to the self-report questionnaire. AMS was diagnosed according to a recent gain in altitude, the presence of headaches and at least one of the following symptoms: gastrointestinal (GI) upset, fatigue, dizziness or insomnia. To study, retrospectively, whether HRV changes could have predicted AMS at higher altitudes, we divided the study group into two groups: (1) If the LLS was ≥ 3 at any altitude the subject was assigned to the AMS group, and (2) If the LLS was ≤ 2 at every altitude measured, the subject was assigned to the no-AMS group.

## Statistical analysis

Continuous values are presented as means ± standard deviation (SD). The frequency of parameters was assessed by *x*^2^ tests with Yates' correction. Differences between AMS and no-AMS groups were compared by nonparametric *t*-test. Correlations between the LLS and clinical and autonomic variables were assessed by Pearson's correlation. In all tests *p* ≤ 0.05 was considered significant.

## Results

**Figure 1:**
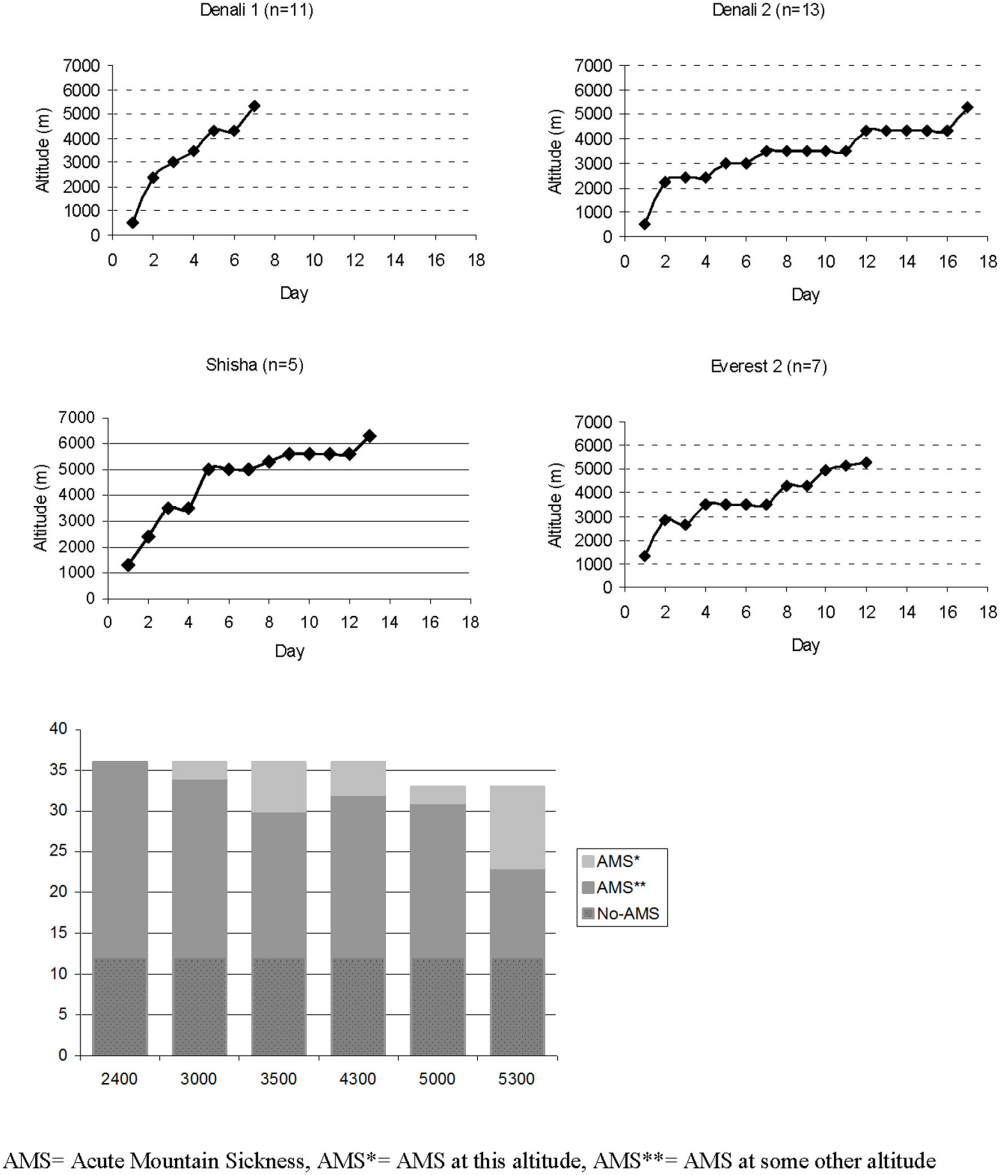
**Ascent profiles of different expeditions and number of AMS cases at different altitudes.** Three climbers descended after 4300 m because of AMS. Measurements were made daily up to 5300 m except on Shisha Pangma, where measurements were made up to 5600 m. AMS, Acute Mountain Sickness; AMS^*^, AMS at this altitude; AMS^**^, AMS at some other altitude.

In total, 36 subjects were studied up to the altitude of 4300 m. Three discontinued because of AMS and the remaining 33 continued up to 5000 and 5300 m altitudes. Five made all measurements at 5600 and one at 6300 m altitude. The Denali 1 and Shisha expeditions reached the altitude of 5000 m in 7 days (*n* = 16) and the Denali 2 and Everest expeditions took 11–17 days to reach the same altitude (*n* = 20) (Figure 1). Acute mountain sickness developed in 24 out of 36 (66%) subjects at some altitude between 3000 and 5600 m, including both women and 22 of 34 men, while 12 men (34%) did not get AMS at any altitude (no-AMS group). There were 11 AMS cases in the faster group and 13 AMS cases in the slower group. The groups did not differ in age, BMI or $\dot{V}{\text{O}}_{2\max}$. The descriptive data of participants and ascents for each expedition are presented in Table 1 and Figure 1.

**Table 1:** **Demographic data (age, BMI, and $\dot{V}{\text{O}}_{2\max}$) of the study population**.

**Characteristic**	**no-AMS (*n* = 12)**	**AMS at some altitude (*n* = 24)**	**All (*n* = 36)**
Age (years)^*^	30 ± 5	33 ± 7	32 ± 6
BMI (kg/m^2^)	24 ± 1	25 ± 4	25 ± 3
$\dot{V}{\text{O}}_{2\max}$ (ml/kg/min)	60 ± 4	60 ± 9	59 ± 7

There were no statistically significant differences in the basic characteristics between no-AMS and AMS groups.

*Values are mean ± SD. BMI, body mass index;* $\dot{V}{\text{O}}_{2\max}$, *maximal oxygen uptake.*

During this study, the daily ascent rates were mostly higher than what is generally recommended (300 m/day) but quite common for these mountains (range 400–1500 m). In the no-AMS group, RMSSD_2 min_, LF_2 min_ and HF_2 min_ increased in the first few days of the ascent. Above 3500 m all HRV parameters decreased while HR increased.

The number of AMS cases at different altitudes is presented in Figure 1. Among those who suffered from AMS, LLS scores varied between 3 and 8. Most of the AMS cases occurred at 5300 m (*n* = 10), and the prevalence of AMS increased significantly with altitude and fast ascent rate on the day before onset (both *p* < 0.001) (Figure 1). Headache and difficulty in sleeping were the most frequent symptoms of AMS followed by GI symptoms, fatigue, and dizziness.

**Figure 2:**
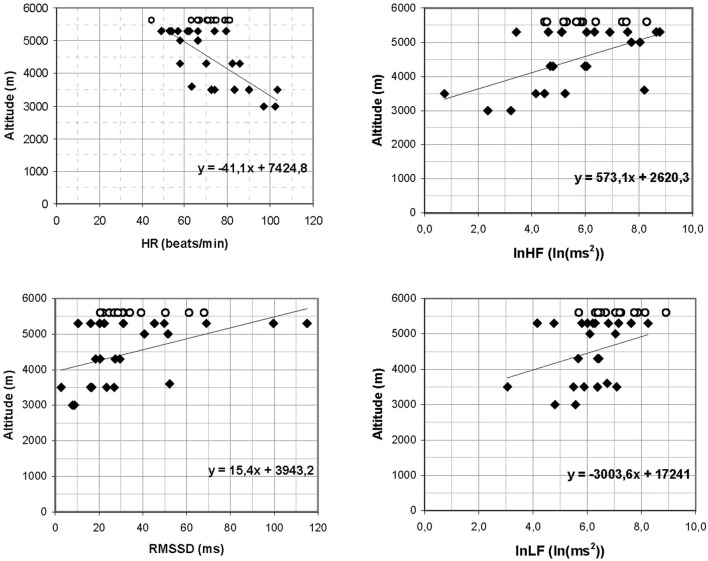
**Negative correlation between HR and positive correlation between lnHF, lnLF, and RMSSD at 2400 m altitude and the lowest altitude at which a climber got AMS (HR *r* = −0.743, *p* < 0.01; lnHF *r* = 0.558, *p* < 0.05, lnLF *r* = 0.302, *p* > 0.05, RMSSD *r* = 0.495, *p* < 0.05).** no-AMS subjects datapoints are added at 5600 m level.

**Figure 3:**
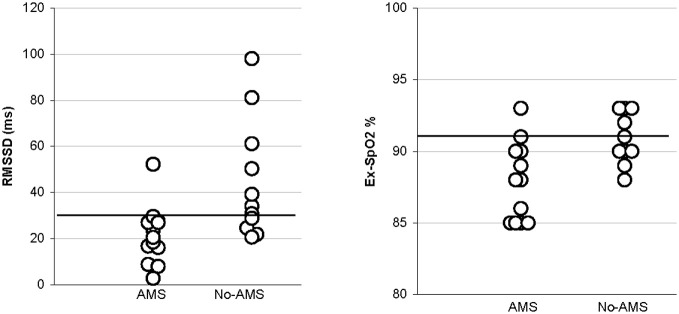
**Scattergram showing the distribution of values of RMSSD and Ex-SpO_2_ measured in 24 study subjects at 2400 m in AMS and no-AMS.** The cut-off lines RMSSD_2min_ ≤ 30 ms and Ex-SpO_2_ ≤ 91% are for 92% sensitivity for AMS.

At 2400 m altitude, RMSSD_2 min_ and HF_2 min_ were lower among those climbers who got AMS at lower altitudes (3000–4300 m) (*n* = 12) than in those who got AMS 3–7 days later at higher altitude (≥5000 m) (*n* = 12) or not at all (*n* = 12) (Table 2). HR_2 min_, lnHF_2 min_ and RMSSD_2 min_ at 2400 m correlated with the lowest altitude at which a climber suffered AMS (AMS altitude) (Figure 2). There were no differences between R-SpO_2_ at 2400 m and later onset of AMS, but Ex-SpO_2_ was statistically higher in the no-AMS than the AMS group at 3000–4300 m (*p* < 0.01) (95% CI 3 (1–5) and in the AMS at ≥ 5000 m group (*p* < 0.05). However, Ex-SpO_2_ did not correlate with the AMS altitude (*r* = −0.028). At the altitude of 2400 m RMSSD_2 min_ ≤ 30 ms and Ex-SpO_2_ ≤ 91% both had 92% sensitivity for AMS at 3000–4300 m if the ascent continued without extra acclimatization days (Figure 3). The sensitivity, specificity, positive, and negative predicative values for chosen cut-off values are presented in Table 3.

**Table 2:** **Resting heart rate (HR) and HRV parameters at 2400 m among the climbers who subsequently developed AMS at two different altitude ranges, and those who had no subsequent AMS**.

	**no-AMS (*n* = 12)**	**AMS at 3000–4300 m (*n* = 12)**	**AMS at ≥ 5000 m (*n* = 12)**
HR (beats/min)	70 ± 9	82 ± 15^*^	62 ± 8^*^^†^
RMSSD (ms)	43 ± 25	21 ± 13^*^	48 ± 32^†^
lnLF (ms^2^)	7 ± 1	6 ± 1^‡^	6 ± 1
lnHF (ms^2^)	6 ± 1	5 ± 2^*^	7 ± 2^†^
LF/HF	5 ± 4	6 ± 6	1.2 ± 1.1^‡^^†^
R-SpO_2_	94 ± 1	93 ± 2	94 ± 2
Ex-SpO_2_	91 ± 2	88 ± 3^‡^	89 ± 3^*^

Values are presented in mean ± SD. (

* p < 0.05,

‡ p < 0.01, difference between AMS 3500–4300 m vs. no-AMS groups and AMS ≥ 5000 m vs. no-AMS groups,

† p < 0.05, difference between AMS 3500–4300 m vs. AMS ≥ 5000 m).

**Table 3:** **Sensitivity and specificity of chosen parameters at 2400 m altitude for AMS at 3000–4300 m**.

	**Sensitivity (%)**	**Specificity (%)**	**Positive predicative value (%)**	**Negative predicative value (%)**	**95% confidence interval**
RMSSD_2 min_ ≤ 30	92	58	61	83	22(5–39)
Ex-SpO_2_ ≤ 91 %	92	40	65	80	3 (1–5)

## Discussion

Our most important finding was that subjects susceptible to AMS had lower HRV before the clinical manifestations of AMS than those who acclimatized well and did not get AMS. This is a new finding and offers fascinating options for predicting AMS at high altitude in field conditions.

The diagnosis of AMS is clinical, but some specific changes in cardiac autonomic function measured by HRV have been estimated to be more sensitive in the detection of the early signs of AMS than clinical symptoms alone at high altitudes (Saito et al., 2005). HRV is reported to decrease in absolute units (Hughson et al., 1994; Bernardi et al., 1998; Kanai et al., 2001) and LF/HF mostly increased when subject got AMS (Loeppky et al., 2003; Lanfranchi et al., 2005; Chen et al., 2008; Huang et al., 2010). There are, however, also opposite findings reported about HRV (Koehle et al., 2010). However, studies conducted in the field at high and extreme altitudes (>5000 m) are still lacking.

During mild sympathetic activation, the observed increase in HR has been reported to be associated with an increase in LF power (Elghozi and Julien, 2007). However, with more intense sympathetic stimulation, the increase in HR was reported to be associated with an overall decrease in HRV, including its LF component. Our present findings on changes in HRV values at extremely high altitudes support this concept.

At 2400 m the no-AMS group had higher HRV and lower HR_2 min_ than the AMS group. The differences are not related to differences in physical fitness in the AMS and no-AMS groups because the basic maximum oxygen uptake did not differ significantly between the groups (59 ± 11 vs. 60 ± 4, *p* > 0.05). Furthermore, clear changes did not come in connection with the ascent. However, in the no-AMS group HF_2 min_ tended to be lower, while LF_2 min_ tended to be higher than in the AMS group. The physiologic significance of these findings remains uncertain, but they do not directly support the earlier assumption that AMS is simply connected with higher cardiac sympathetic activity. Rather, it seemed that a rise in cardiac sympathetic activity would have been a protective phenomenon against altitude illness. However, altitude illness is a dynamic process. It may be that as the AMS proceeds, HRV could further decrease. In other words, changes in HRV were related to higher altitudes in all climbers, but also notably to acclimatization and especially to failure to acclimatize.

The present findings are still generally in agreement with those of earlier studies and provide further insights into the usefulness of HRV in the prediction of AMS under field conditions. Several studies have shown that of SpO_2_ measurements at rest (R-SpO_2_) and immediately after exercise (Ex-SpO_2_) are predictors of AMS (Roach et al., 1998; Saito et al., 2005; Burtscher et al., 2008; Karinen et al., 2010). Our study supports this. At 2400 m altitude the Ex-SpO_2_ was statistically higher in no-AMS group than AMS 3000–4300 group (*p* < 0.01) and AMS ≥ 5000 m group (*p* < 0.05). However, the SpO_2_ measurements made by pulse oximetry under field conditions may be susceptible to many disruptive factors (temperature, bright light, cold fingers etc.) (Luks and Swenson, 2011). Therefore, it is desirable to have an another parameter(s) to follow-up for AMS susceptibility. Our findings support the possibility that HRV could be used along with commonly measured physiological parameters, HR and SpO_2_, clinical status and Lake Louise questionnaire for AMS prediction in the short time period at moderate altitudes. The altitude of 5000 m was reached mainly 1–2 weeks after the measurements at 2400 m and the ascent rates per day were high just before reaching this altitude (700–1500 m/day). This may explain better the high prevalence of AMS at 5000–5300 m than a predominant vagal modulation at rest at 2400 m.

The limitations of the present study include the relatively small study group (*n* = 33) at extreme altitude (>5000 m). The correlations between HRV parameters and AMS are rather weak. Thus, further studies with larger populations are needed to verify the early changes in HRV between subjects with and without AMS and/or other high-altitude illnesses, especially at extreme altitudes, where the consequences of AMS, HACE, and HAPE may dramatic. The length of measurements at high altitude may have been too short to fully observe trends of changes in HRV variables between subjects with and without AMS or to capture other HRV measures that could reflect more severe periodic breathing. The differences in ascent rates, the places studied and the altitudes studied may in part explain the differences between the different results. Environmental and/or cardiovascular effects of previous exercise may have been factors in the autonomic patterns observed in subjects with AMS in our study. However, subjects with and without AMS were studied under the same field conditions and after similar recovery times as regards ascent. The ascent rates during the expeditions were normal and widely used in the mountains. Therefore, exercise and environmental factors may not explain the differences in autonomic cardiovascular function observed in association with AMS in our study. HRV is also partially influenced by respiratory rate and depth (Task Force, 1996; Penttilä et al., 2001). In the HRV frequency spectrum, too, power in the entire frequency area, not just the HF band, is decreased if the respiration rate increases (Task Force, 1996). Normally respiration rates will increase somewhat at high altitude, but we do not know if the respiration rate is higher in subjects with AMS than in subjects with no AMS. In this study, the subjects were breathing spontaneously and some of the subjects may have had breathing rate close to or below 0.15 Hz, which is the lowest boundary for the HF band. For this reason, if altitude or AMS changed respiratory HRV, it cannot be exclusively judged by changes in HF or LF/HF. Unfortunately, we do not have sea-level HRV data for all subjects, so we cannot estimate whether those climbers whose basic level of HF is higher could acclimatize better than those whose HF level is lower at sea-level.

Periodic breathing has reported to exist at high altitude mainly during the sleep, but also in some extent during wake (Insalaco et al., 1996; Fan et al., 2012). Because the breathing frequency was not measured in our study, we cannot exclude it totally (McMullen et al., 2012). However, the visual analysis of RRI tachograms, AR spectrum and Poincare plots do not support the existence of periodic breathing during the measurements. The measurement periods were 2 min so we can assume that any significant periodic breathing was not seen when lying down during this measurement period.

The strengths of the present study are that the rate of ascent, altitude of origin and time of day of testing were known. Also, within each group every climber had a similar diet, they were relatively homogenous in their exercise capacity and they climbed the same route on mountains over a short period of time with essentially the same snow and weather conditions. Barometric pressure varied from day to day but during measurements it was stable.

In conclusion, at 2400 m decreased RMSSD_2 min_ and HF_2 min_ both predicted AMS in a few days if the ascent continued without rest days. Autonomic cardiac response to increasing altitude could be a low-cost non-invasive test to predict impending AMS and to help distinguish those who are at risk for AMS and those who are acclimatizing well. The trigger values typical for impending AMS await further studies.

### Conflict of interest statement

The authors declare that the research was conducted in the absence of any commercial or financial relationships that could be construed as a potential conflict of interest.
